# The Combination of Bromelain and Acetylcysteine (BromAc) Synergistically Inactivates SARS-CoV-2

**DOI:** 10.3390/v13030425

**Published:** 2021-03-06

**Authors:** Javed Akhter, Grégory Quéromès, Krishna Pillai, Vahan Kepenekian, Samina Badar, Ahmed H. Mekkawy, Emilie Frobert, Sarah J. Valle, David L. Morris

**Affiliations:** 1 Department of Surgery, St. George Hospital, Sydney, NSW 2217, Australia; Javed.Akhter@health.nsw.gov.au (J.A.); vahan.kepenekian@chu-lyon.fr (V.K.); samina.badar@unsw.edu.au (S.B.); z3170073@ad.unsw.edu.au (A.H.M.); sarah.valle@mucpharm.com (S.J.V.); 2 Mucpharm Pty Ltd., Sydney, NSW 2217, Australia; panthera6444@yahoo.com.au; 3 CIRI, Centre International de Recherche en Infectiologie, Team VirPatH, Univ Lyon, Inserm, U1111, Université Claude Bernard Lyon 1, CNRS, UMR5308, ENS de Lyon, F-69007 Lyon, France; gregory.queromes@univ-lyon1.fr (G.Q.); emilie.frobert@chu-lyon.fr (E.F.); 4 Hospices Civils de Lyon, EMR 3738 (CICLY), Lyon 1 Université, F-69921 Lyon, France; 5 St. George & Sutherland Clinical School, University of New South Wales, Sydney, NSW 2217, Australia; 6 Laboratoire de Virologie, Institut des Agents Infectieux (IAI), Hospices Civils de Lyon, Groupement Hospitalier Nord, F-69004 Lyon, France

**Keywords:** SARS-CoV-2, Bromelain, Acetylcysteine, BromAc, drug repurposing

## Abstract

Severe acute respiratory syndrome coronavirus (SARS-CoV-2) infection is the cause of a worldwide pandemic, currently with limited therapeutic options. The spike glycoprotein and envelope protein of SARS-CoV-2, containing disulfide bridges for stabilization, represent an attractive target as they are essential for binding to the ACE2 receptor in host cells present in the nasal mucosa. Bromelain and Acetylcysteine (BromAc) has synergistic action against glycoproteins by breakage of glycosidic linkages and disulfide bonds. We sought to determine the effect of BromAc on the spike and envelope proteins and its potential to reduce infectivity in host cells. Recombinant spike and envelope SARS-CoV-2 proteins were disrupted by BromAc. Spike and envelope protein disulfide bonds were reduced by Acetylcysteine. In in vitro whole virus culture of both wild-type and spike mutants, SARS-CoV-2 demonstrated a concentration-dependent inactivation from BromAc treatment but not from single agents. Clinical testing through nasal administration in patients with early SARS-CoV-2 infection is imminent.

## 1. Introduction

The recently emergent severe acute respiratory syndrome coronavirus 2 (SARS-CoV-2) is the causative agent of coronavirus disease 2019 (COVID-19), which can range from asymptomatic to severe and lethal forms with a systemic inflammatory response syndrome. As of 21 February 2021, over 111 million confirmed cases have been reported, with an estimated overall mortality of 2.2% [1]. There are currently few therapeutic agents proven to be beneficial in reducing early- and late-stage disease progression [2]. While there are fortunately many vaccine candidates, their widespread availability for vaccination may not be immediate, the length of immune protection may be limited [3,4], and the efficacy of the vaccines may be reduced by novel SARS-CoV-2 variants. The continued exploration of effective treatments is therefore still needed.

Structurally, SARS-CoV-2 contains surface spike proteins, membrane proteins, and envelope proteins, as well as internal nucleoproteins that package the RNA. The spike protein is a homotrimer glycoprotein complex with different roles accomplished through dynamic conformational modifications, based in part on disulfide bonds [5]. It allows the infection of target cells by binding to the human angiotensin-converting enzyme (ACE2) receptors, among others, which triggers proteolysis by transmembrane protease serine 2 (TMPRSS2), furin, and perhaps other proteases, leading to virion and host cell membrane fusion [6,7].

The entry of viruses into mammalian cells, or “virus internalization”, is a key mechanism of enveloped virus infection and is based on dynamic conformational changes of their surface glycoproteins, namely, as mediated by disulfide bond reduction and regulated by cell surface oxydoreductases and proteases [5,8,9,10,11]. SARS-CoV-2 entry into host cells has been shown to start with destabilization of the spike protein through allosteric mechanical transition, which induces a conformational change from the closed “down” state to open “up” state of the receptor binding domain (RBD) of the spike protein [12,13]. The conformational changes of RBD and virus binding are induced by TMPRSS2 or Cathepsin L, which trigger the transition from the pre-fusion to post-fusion state [5,12,13]. The energy liberated by disulfide bond reduction increases protein flexibility, which is maximal when the reduced state is complete [8], thus allowing the fusion of host–virus membranes, which is otherwise impossible due to the repulsive hydration forces present before reduction [5].

Bromelain is extracted mainly from the stem of the pineapple plant (*Ananas comosus*) and contains a number of enzymes that give it the ability to hydrolyze glycosidic bonds in complex carbohydrates [14]. Previous studies have indicated that Bromelain removes the spike and hemagglutinin proteins of Semliki Forest virus, Sindbis virus, mouse gastrointestinal coronavirus, hemagglutinating encephalomyelitis virus, and H1N1 influenza viruses [15,16]. As a therapeutic molecule, it is used for debriding burns. Acetylcysteine is a powerful antioxidant that is commonly nebulized into the airways for mucus accumulation and is also used as a hepatoprotective agent in paracetamol overdose. Most importantly in the present context, Acetylcysteine reduces disulfide bonds [17]. Moreover, the association of the spike and envelope proteins by their respective triple cysteine motifs warrants the hypothesis of impacting virion stability following disulfide bridge disruption by the action of Acetylcysteine [18]. The combination of Bromelain and Acetylcysteine (BromAc) exhibits a synergistic mucolytic effect that is used in the treatment of mucinous tumors [19,20] and as a chemosensitizer of several anticancer drugs [21]. These different actions are due to the ability of BromAc to unfold the molecular structures of complex glycoproteins, thus allowing binding to occur because of the high affinity between RBD and ACE2. 

Therefore, in the current study we set out to determine whether BromAc can disrupt the integrity of SARS-CoV-2 spike and envelope proteins and subsequently examine its inactivation potential against in vitro replication of two viral strains, including one with a spike mutant alteration of the novel S1/S2 cleavage site.

## 2. Materials and Methods

### 2.1. Materials

Bromelain API was manufactured by Mucpharm Pty Ltd (Kogarah, Australia) as a sterile powder. Acetylcysteine was purchased from Link Pharma (Cat# AUST R 170803; Warriewood, Australia). The recombinant SARS-COV-2 spike protein was obtained from SinoBiological (Cat# 40589-V08B1; Beijing, China). The recombinant envelope protein was obtained from MyBioSource (Cat# MBS8309649; San Diego, CA, USA). All other reagents were from Sigma Aldrich (St. Louis, MO, USA). 

### 2.2. Recombinant Spike and Envelope Gel Electrophoresis 

The spike or envelope proteins were reconstituted in sterile distilled water according to the manufacturer’s instructions, and aliquots were frozen at −20 °C. Two and a half micrograms of spike or envelope protein were incubated with 50 or 100 µg/mL Bromelain, 20 mg/mL Acetylcysteine, or a combination of both in Milli-Q water. The control contained no drugs. The total reaction volume was 15 µL each. After 30 min incubation at 37 °C, 5 µL of sample buffer was added into each reaction. A total of 20 µL of each reaction was electrophoresed on an SDS-PAGE (Cat# 456-1095; Bio-Rad Hercules, CA, USA). The gels were stained using Coomassie blue.

### 2.3. UV Spectral Detection of Disulfide Bonds in Spike and Envelope Proteins

The method of Iyer and Klee for the measurement of the rate of reduction of disulfide bonds has been used to detect disulfide bonds in spike and envelope proteins [22]. The recombinant SARS-CoV-2 spike protein at a concentration of 3.0 µg/mL in phosphate-buffered saline (PBS) (pH 7.0) containing 1 mM ethylenediaminetetraacetic acid (EDTA) was incubated with 0, 10, 20, 40, and 50 µL of Acetylcysteine (0.5 M), agitated at 37 °C for 30 min followed by equivalent addition of Dithiothreitol (DTT) (0.5 M), and agitated for a further 30 min at 37 °C. The spike protein was incubated in parallel only with DTT (0.5 M) as before without any Acetylcysteine and agitated at 37 °C for 30 min. The absorbance was then read at 310 nm. UV spectral detection of disulfide bonds in the envelope protein was performed in a similar manner.

### 2.4. SARS-CoV-2 Whole Virus Inactivation with BromAc 

Fully respecting the World Health Organization (WHO) interim biosafety guidance related to the coronavirus disease, the SARS-CoV-2 whole virus inactivation tests were carried out with a wild-type (WT) strain representative of early circulating European viruses (GISAID accession number EPI_ISL_578176). A second SARS-CoV-2 strain (denoted as ∆S), reported through routine genomic surveillance in the Auvergne-Rhône-Alpes region of France, was added to the inactivation tests due to a rare mutation in the spike S1/S2 cleavage site and its culture availability in the laboratory (GISAID accession number EPI_ISL_578177).

These tests were conducted with incremental concentrations of Bromelain alone (0, 25, 50, 100, and 250 µg/mL), Acetylcysteine alone (20 mg/mL), and the cross-reaction of the different Bromelain concentrations combined with a constant 20 mg/mL Acetylcysteine formulation, against two virus culture dilutions at 10^5.5^ and 10^4.5^ TCID50/mL. Following 1 h of drug exposure at 37 °C, all conditions, including the control, were diluted 100-fold to avoid cytotoxicity, inoculated in quadruplicate on confluent Vero cells (CCL-81; ATCC©, Manassas, VA, USA), and incubated for 5 days at 36 °C with 5% CO_2_. Cells were maintained in Eagle’s minimal essential medium (EMEM) with 2% Penicillin-Streptomycin, 1% L-glutamine, and 2% inactivated fetal bovine serum. Results were obtained by daily optical microscopy observations, an end-point cell lysis staining assay, and reverse-transcriptase polymerase chain reaction (RT-PCR) of supernatant RNA extracts. Briefly, the end-point cell lysis staining assay consisted of adding Neutral Red dye (Merck KGaA, Darmstadt, Germany) to cell monolayers, incubating at 37 °C for 45 min, washing with PBS, and adding citrate ethanol before optical density (OD) was measured at 540 nm (Labsystems Multiskan Ascent Reader, Thermo Fisher Scientific, Waltham, MA, USA). OD was directly proportional to viable cells, so a low OD would signify important cell lysis due to virus replication. In addition, RNA from well supernatants was extracted by the semi-automated eMAG^®^ workstation (bioMérieux, Lyon, FR), and SARS-CoV-2 RdRp IP2-targeted RdRp Institute Pasteur RT-PCR was performed on a QuantStudio™ 5 System (Applied Biosystems, Thermo Fisher Scientific, Foster City, CA, USA). Log_10_ reduction values (LRV) of viral replication were calculated by the difference between treatment and control wells per condition divided by 3.3 (as 1 log_10_ ≈ 3.3 PCR Cycle thresholds (Ct)).

### 2.5. Replication Kinetics by Real-Time Cell Analysis

To compare the in vitro replication capacity of both WT and ∆S SARS-CoV-2 strains, replication kinetics were determined by measuring the electrode impedance of microelectronic cell sensors on the xCELLigence Real-Time Cell Analyzer (RTCA) DP Instrument (ACEA Biosciences, Inc., San Diego, CA, USA). Vero cells were seeded at 20,000 cells per well on an E-Plate 16 (ACEA Biosciences, Inc., San Diego, CA, USA) and incubated with the same media conditions as described previously at 36 °C with 5% CO2. After 24 h, SARS-CoV-2 culture isolates were inoculated in triplicate at a multiplicity of infection of 10−2. Mock infections were performed in quadruplicate. Electronic impedance data (cell index) were continuously collected at 15-min intervals for 6 days. Area under the curve analysis of normalized cell index, established at time of inoculation, was then calculated at 12-h intervals. At each interval, cell viability was determined by normalizing against the corresponding cell control. Tukey multiple comparison tests were used to compare each condition on GraphPad Prism (software version 9.0; San Diego, CA, USA).

## 3. Results

### 3.1. Alteration of SARS-CoV-2 Spike and Envelope Proteins

**Figure 1:**
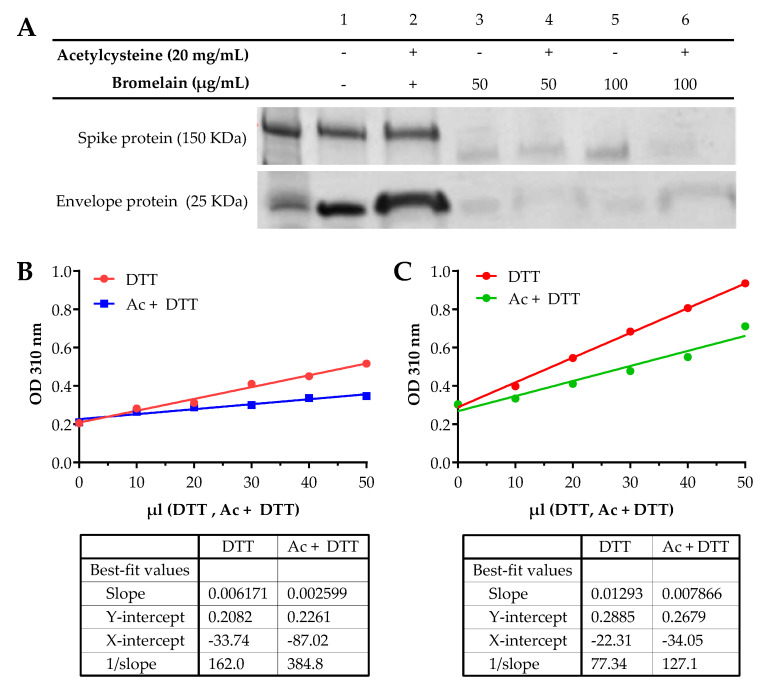
(**A**) Bromelain and Acetylcysteine present a synergistic effect on severe acute respiratory syndrome coronavirus (SARS-CoV-2) spike and envelope protein destabilization. SDS-PAGE of the recombinant SARS-CoV-2 spike protein S1 + S2 subunits (150 kDa) and envelope protein (25 kDa). Proteins were treated with 20 mg/mL Acetylcysteine alone, 100 and 50 µg/mL Bromelain alone, and a combination of 100 and 50 µg/20 mg/mL BromAc. (**B**) Disulfide reduction of recombinant SARS-CoV-2 spike protein by Acetylcysteine. The differential assay between Acetylcysteine (Ac) and Dithiothreitol (DTT) for the reduction of disulfide bonds found on the spike protein indicates that Acetylcysteine reduces 42% of the disulfide bonds before the addition of DTT. The remaining bonds are reduced by DTT to produce the chromogen detected at 310 nm. (**C**) Disulfide reduction of recombinant SARS-CoV-2 envelope protein by Acetylcysteine. The differential assay between Acetylcysteine (Ac) and Dithiothreitol (DTT) for the reduction of disulfide bonds found on the envelope protein indicates that Acetylcysteine reduces 40% of the bonds before the addition of DTT.

Treatment of the spike protein with Acetylcysteine alone did not show any alteration of the protein, whereas concentrations of Bromelain at 50 and 100 µg/mL and BromAc at 50 and 100 µg/20 mg/mL resulted in protein alteration (Figure 1A). Treatment with Acetylcysteine on the envelope protein did not alter the protein, whereas treatment with Bromelain at 50 and 100 µg/mL and BromAc at 50 and 100 µg/20 mg/mL also resulted in near complete and complete fragmentation, respectively (Figure 1A).

### 3.2. UV Spectral Detection Demonstrates the Alteration of Disulfide Bonds in Spike and Envelope Proteins 

The comparative reduction of disulfide bonds on the spike protein between DTT alone and DTT with Acetylcysteine demonstrated a 42% difference (Figure 1B), based on the slope of the graphs [0.002599/0.006171 (100) = 42 %]. Acetylcysteine was thus able to reduce 58% of the disulfide linkages in the sample, after which the remaining disulfide bonds were reduced by DTT to produce the chromogen that was monitored in the spectra. Similarly, the differential assay between Acetylcysteine and DTT for the reduction of disulfide bonds found in the envelope protein [0.007866/0.01293 (100) = 60%] indicates that Acetylcysteine reduces 40% of the disulfide bonds before the addition of DTT (Figure 1C).

### 3.3. In Vitro SARS-CoV-2 Inactivating Potential of Bromelain, Acetylcysteine, and BromAc

**Figure 2:**
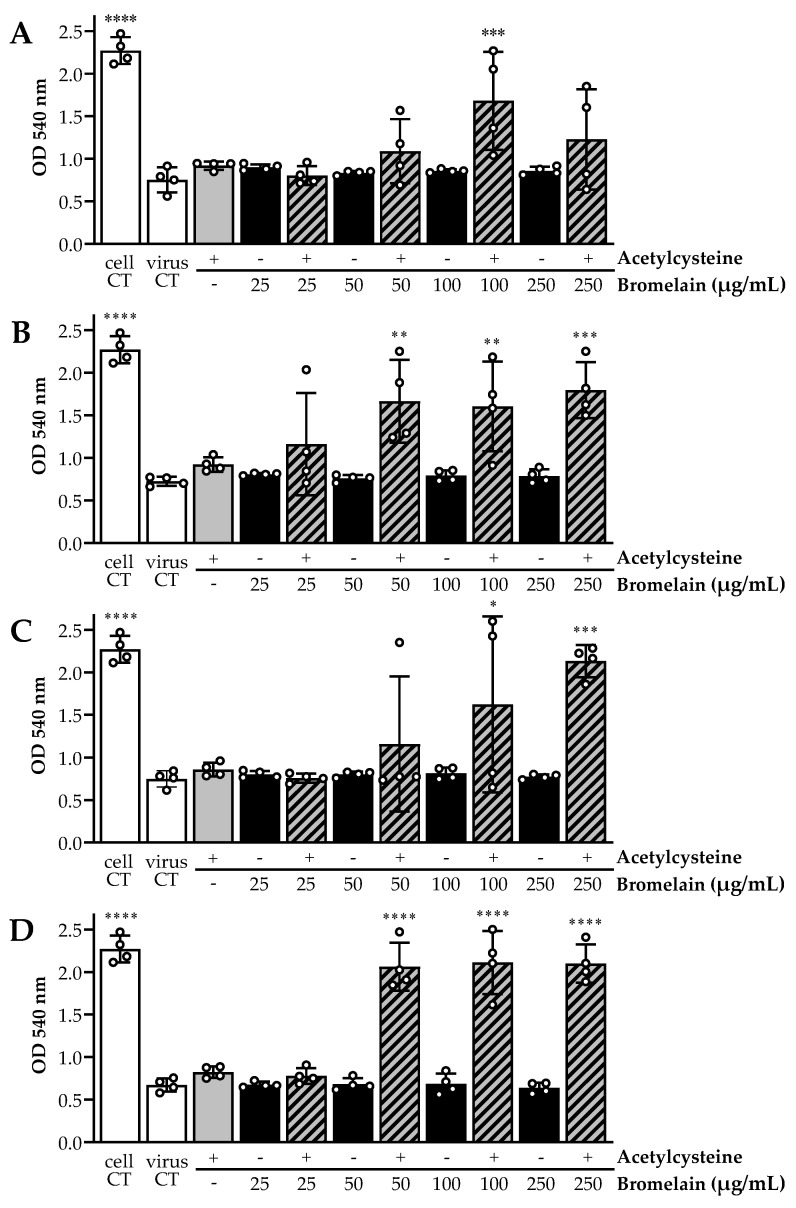
Cell lysis assays demonstrated in vitro inactivation potential of Acetylcysteine and Bromelain combined (BromAc) against SARS-CoV-2. Cell viability was measured by cell staining with Neutral Red, where optical density (OD) is directly proportional to viable cells. Low OD would signify important cell lysis due to virus replication. The wild-type (WT) SARS-CoV-2 strain at 5.5 and 4.5 log_10_TCID_50_/mL titers (**A** and **B**, respectively) showed no inhibition of cytopathic effect (CPE) for single agent treatment, compared to the mock treatment virus control condition. BromAc combinations were able to inhibit CPE, compared to the mock infection cell controls. Treatment of a SARS-CoV-2 spike protein variant (∆S) with a mutation at the S1/S2 junction at 5.5 and 4.5 log_10_TCID_50_/mL titers (**C** and **D**, respectively) showed similar results. Bars represent the average of each quadruplicate per condition, illustrated by white circles. Ordinary one-way ANOVA was performed, using the mock treatment virus control as the control condition (**** *p* < 0.0001, *** *p* < 0.0005, ** *p* < 0.003, and * *p* < 0.05).

For both SARS-CoV-2 strains tested, the untreated virus controls at 10^5.5^ and 10^4.5^ TCID_50_/mL yielded typical cytopathic effects (CPE), and no cytotoxicity was observed for any of the drug combinations on Vero cells. Optical CPE results were invariably confirmed by end-point Neutral Red cell staining. Overall, Bromelain and Acetylcysteine treatment alone showed no viral inhibition, all with CPE comparable to virus control wells, whereas BromAc combinations displayed virus inactivation in a concentration-dependent manner (Figure 2). Treatment on 10^4.5^ TCID_50_/mL virus titers (Figure 2B,D) yielded more consistent inhibition of CPE for quadruplicates than on 10^5.5^ TCID_50_/mL virus titers (Figure 2A,C).

**Figure 3:**
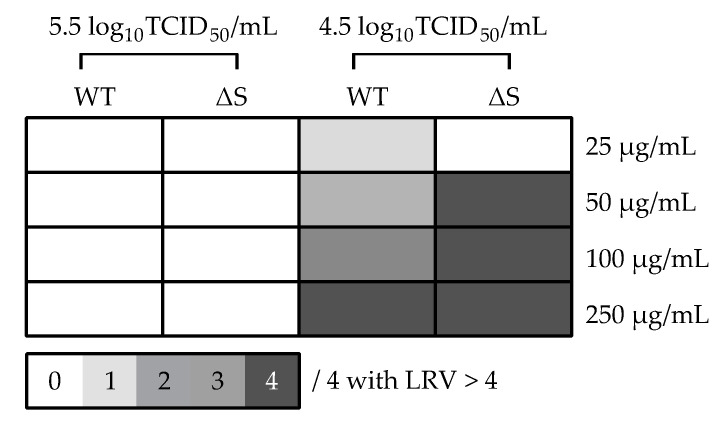
Threshold matrix of log_10_ reduction values (LRV) of in vitro virus replication 96 h after BromAc treatment on WT and ∆S SARS-CoV-2 strains at 5.5 and 4.5 log_10_TCID_50_/mL titers. LRV were calculated with the following formula: LRV = (RT-PCR Ct of treatment—RT-PCR Ct virus control)/3.3; as 1 log10 ≈ 3.3 Ct. The color gradient matrix displays the number of quadruplicates per condition yielding an LRV > 4, corresponding to a robust inactivation according to the WHO. WT = wild-type; ∆S = S1/S2 spike mutant.

Based on the virus inactivation guidelines established by the WHO, a robust and reliable process of inactivation will be able to reduce replication by at least 4 logs [Log_10_ reduction value (LRV) = (RT-PCR Ct treatment – RT-PCR Ct control)/3.3; as 1 log_10_ ≈ 3.3 Ct]. As such, RT-PCR was performed on the RNA extracts to directly measure virus replication. For the wild-type (WT) strain at 10^4.5^ TCID_50_/mL, successful LRV > 4 were observed with 1 out of 4 wells, 2 out of 4 wells, 3 out of 4 wells, and 4 out of 4 wells for 25, 50, 100 and 250 µg/20 mg/mL BromAc, respectively (Figure 3). It is worth noting that at 10^5.5^ TCID_50_/mL, LRV were slightly below the threshold at, on average, 3.3, with 3 out of 4 wells and 2 out of 4 wells for 100 and 250 µg/20 mg/mL BromAc, respectively (Table 1). For the spike protein mutant (∆S) at 10^4.5^ TCID_50_/mL, no successful LRV > 4 was observed for 25 µg/20 mg/mL BromAc, but it was observed in 4 out of 4 wells for 50, 100, and 250 µg/20 mg/mL BromAc (Figure 3). Of note, at 10^5.5^ TCID_50_/mL, LRV were slightly below the threshold at, on average, 3.2, with 1 out of 4 wells, 2 out of 4 wells, and 4 out of 4 wells for 50, 100, and 250 µg/20 mg/mL BromAc, respectively (Table 1). Overall, in vitro inactivation of both SARS-CoV-2 strains’ replication capacity was observed in a dose-dependent manner, most strongly demonstrated at 100 and 250 µg/20 mg/mL BromAc against 10^4.5^ TCID_50_/mL of virus.

**Figure 4:**
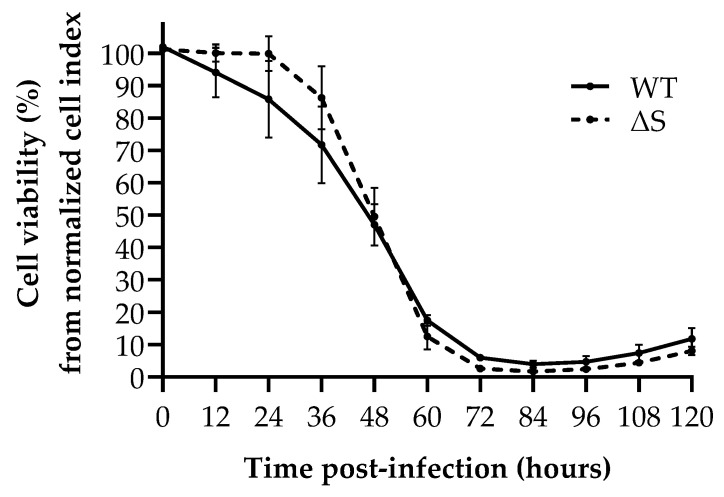
SARS-CoV-2 replication capacity of WT and ∆S SARS-CoV-2 measured by Real-Time Cell Analysis. Data points correspond to area under the curve analysis of normalized cell index (electronic impedance of RTCA established at time of inoculation) at 12-h intervals. Cell viability was then determined by normalizing against the corresponding cell control. WT = wild-type; ∆S = S1/S2 spike mutant.

Real-time cell analysis demonstrated comparable replication kinetics for both WT and ∆S SARS-CoV-2 strains (Figure 4). No significant difference in cell viability was observed between WT and ∆S at any time point. From 48 h post-infection, WT and ∆S cell viability were significantly different compared to the mock infection (*p* < 0.05).

## 4. Discussion

The combination of Bromelain and Acetylcysteine, BromAc, synergistically inhibited the infectivity of two SARS-CoV-2 strains cultured on Vero cells. Protein confirmation and its molecular properties are dependent on its structural and geometric integrity, which are dependent on both the peptide linkages and disulfide bridges. Acetylcysteine, as a good reducing agent, tends to reduce the disulfide bridges and hence alter the molecular properties of most proteins. This property has been widely exploited in the development of several therapies (chronic obstructive pulmonary disease, allergic airways diseases, cystic fibrosis, pseudomyxoma peritonei, etc.) [20,23,24,25,26,27]. More recently, Acetylcysteine has been used in the development of therapies for respiratory infections such as influenza and COVID-19 [28,29,30], where the integrity of the spike protein is vital for infection [12,13]. A hypothesized mechanism of action could be the unfolding of the spike glycoprotein and the reduction of its disulfide bonds.

The SARS-CoV-2 spike protein is the cornerstone of virion binding to host cells and hence represents an ideal therapeutic target. A direct mechanical action against this spike protein is a different treatment strategy in comparison to most of the existing antiviral drugs, which prevents viral entry in host cells rather than targeting the replication machinery. BromAc acts as a biochemical agent to destroy complex glycoproteins. Bromelain’s multipotent enzymatic competencies, dominated by the ability to disrupt glycosidic linkages, usefully complement Acetylcysteine’s strong power to reduce disulfide bonds [17]. Amino acid sequence analysis of the SARS-CoV-2 spike glycoprotein identified several predetermined sites where BromAc could preferentially act, such as the S2’ site rich in disulfide bonds [31], together with three other disulfide bonds in RBD [32]. In parallel, the role of the glycosidic shield in covering the spike, which is prone to being removed by BromAc, has been highlighted as a stabilization element of RBD conformation transitions as well as a resistance mechanism to specific immune response [5,33,34]. 

Mammalian cells exhibit reductive functions at their surface that are capable of cleaving disulfide bonds, and the regulation of this thiol-disulfide balance has been proven to impact the internalization of different types of viruses, including SARS-CoV-2 [8,35,36,37,38]. Both ACE2 and spike proteins possess disulfide bonds. When all the spike protein RBD disulfide bonds were reduced to thiols, ACE2 receptor binding to spike protein became less favorable [8]. Interestingly, the reduction of ACE2 disulfide bonds also induced a decrease in binding [8]. Moreover, other reports suggested that Bromelain alone could inhibit SARS-CoV-2 infection in VeroE6 cells through an action on disulfide links [39,40]. As such, the loss of SARS-CoV-2 infectivity observed after pre-treatment with BromAc could be correlated to the cumulative unfolding of the spike and envelope proteins, with a significant reduction of their disulfide bonds by Acetylcysteine, demonstrated in vitro. 

Interestingly, a similar effect of BromAc was observed against both WT and ∆S SARS-CoV-2. The main difference in amino acid sequences between SARS-CoV-2 and previous SARS-CoV is the inclusion of a furin cleavage site between S1 and S2 domains [41]. This distinct site of the spike protein and its role in host spill-over and virus fitness is a topic of much debate [41,42,43,44]. Of note, ∆S, which harbors a mutation in this novel S1/S2 cleavage site and alters the cleavage motif, exhibits no apparent difference in replication capacity compared to the WT strain. The slightly increased sensitivity of ∆S to BromAc treatment is therefore not due to a basal replication bias, but the mutation could perhaps be involved in enhancing the mechanism of action of BromAc. These results would nevertheless suggest that, from a threshold dose, BromAc could potentially be effective on spike mutant strains. This may be a clear advantage for BromAc over specific immunologic mechanisms of a spike-specific vaccination [3,4].

To date, different treatment strategies have been tested, but no molecules have demonstrated a clear antiviral effect. In addition, given the heterogeneous disease outcome of COVID-19 patients, the treatment strategy should combine several mechanisms of action and be adapted to the stage of the disease. Thus, treatment repurposing remains an ideal strategy against COVID-19, whilst waiting for sufficient vaccination coverage worldwide [45,46]. In particular, the development of early nasal-directed treatment prone to decreasing a patient’s infectivity and preventing the progression towards severe pulmonary forms is supported by a strong rationale. Hou et al. demonstrated that the first site of infection is the nasopharyngeal mucosa, with secondary movement to the lungs by aspiration [47]. Indeed, the pattern of infectivity of respiratory tract cells followed ACE2 receptor expression, decreasing from the upper respiratory tract to the alveolar tissue. The ratio for ACE2 was five-fold greater in the nose than in the distal respiratory tract [40]. Other repurposing treatments as a nasal antiseptic have been tested in vitro, such as Povidone-Iodine, which has shown activity against SARS-CoV-2 [48]. In the present study, we showed the in vitro therapeutic potential of BromAc against SARS-CoV-2 with a threshold efficient dose at 100 µg/20 mg/mL. As animal airway safety models in two species to date have exhibited no toxicity (unpublished data), the aim is to test nasal administration of the drug in a phase I clinical trial (ACTRN12620000788976). Such treatment could help mitigate mild infections and prevent infection of persons regularly in contact with the virus, such as health-care workers. 

Although our results are encouraging, there are a number of points to consider regarding this demonstration. Namely, the in vitro conditions are fixed and could be different from in vivo. Any enzymatic reaction is influenced by the pH of the environment, and even more so when it concerns redox reactions such as disulfide bond reduction [9]. The nasal mucosal pH is, in physiological terms, between 5.5 and 6.5 and increases in rhinitis to 7.2–8.3 [49]. Advanced age, often encountered in SARS-CoV-2 symptomatic infections, also induces a nasal mucosa pH increase [49]. Such a range of variation, depending on modifications typically induced by a viral infection, may challenge the efficacy of our treatment strategy. Further in vitro experiments to test various conditions of pH are ongoing, but ultimately, only clinical studies will be able to assess this point. Our experiments were led on a monkey kidney cell line known to be highly permissive to SARS-CoV-2 infectivity. With the above hypothesis of S protein lysis thiol-disulfide balance disruption, BromAc efficacy on SARS-CoV-2 should not be influenced by the membrane protease pattern. Reproducing this experimental protocol with the human pulmonary epithelial Calu-3 cell line (ATCC^®^ HTB-55™) would allow these points to be addressed, as virus entry is TMPRSS2-dependent and pH-independent, as in airway epithelium, while virus entry in Vero cells is Cathepsin L-dependent, and thus pH-dependent [50].

Overall, results obtained from the present study in conjunction with complementary studies on BromAc properties and SARS-CoV-2 characterization reveal a strong indication that BromAc can be developed into an effective therapeutic agent against SARS-CoV-2.

## 5. Conclusions

There is currently no suitable therapeutic treatment for early SARS-CoV-2 aimed at preventing disease progression. BromAc is under clinical development by the authors for mucinous cancers due to its ability to alter complex glycoprotein structures. The potential of BromAc on SARS-CoV-2 spike and envelope proteins stabilized by disulfide bonds was examined and found to induce the unfolding of recombinant spike and envelope proteins by reducing disulfide stabilizer bridges. BromAc also showed an inhibitory effect on wild-type and spike mutant SARS-CoV-2 by inactivation of its replication capacity in vitro. Hence, BromAc may be an effective therapeutic agent for early SARS-CoV-2 infection, despite mutations, and even have potential as a prophylactic in people at high risk of infection.

## Figures and Tables

**Table 1:** Log_10_ reduction values (LRV) of in vitro virus replication 96 h after BromAc treatment on WT and ∆S SARS-CoV-2 strains at 5.5 and 4.5 log_10_TCID_50_/mL titers. LRV were calculated with the following formula: LRV = (RT-PCR Ct of treatment – RT-PCR Ct virus control)/3.3; as 1 log_10_ ≈ 3.3 Ct. Each replicate is described. TCID_50_/mL = Median Tissue Culture Infectious Dose; WT = wild-type; ∆S = S1/S2 spike mutant.

	BromAc (µg/20 mg/mL)	Virus Titer	
		5.5 log_10_TCID_50_/mL	4.5 log_10_TCID_50_/mL
WT	25	0.033 0.104 0.250 0.213	0.463 0.356 4.390 0.173
	50	0.050 0.304 0.446 0.698	0.471 4.378 0.404 4.651
	100	3.415 3.323 0.360 3.313	4.418 4.463 0.423 4.508
	250	0.033 3.423 0.200 3.389	4.496 4.370 4.419 4.506
∆S	25	0.010 0.153 NA 0.414	0.330 0.313 0.172 0.075
	50	3.252 0.297 0.278 0.275	4.762 4.612 4.618 4.571
	100	3.191 3.260 0.210 0.301	6.054 4.518 5.155 4.747
	250	3.287 3.298 3.308 3.308	4.333 4.302 4.410 4.361

## Data Availability

A preprint of this manuscript was archived on www.biorxiv.org (accessed on 31 January 2021) due to the emergency of COVID-19.
